# miRNA Regulation of Cell Phenotype and Parietal Remodeling in Atherosclerotic and Non-Atherosclerotic Aortic Aneurysms: Differences and Similarities

**DOI:** 10.3390/ijms25052641

**Published:** 2024-02-24

**Authors:** Sonia Terriaca, Amedeo Ferlosio, Maria Giovanna Scioli, Francesca Coppa, Fabio Bertoldo, Calogera Pisano, Beatrice Belmonte, Carmela Rita Balistreri, Augusto Orlandi

**Affiliations:** 1 Anatomic Pathology, Policlinico Tor Vergata, 00133 Rome, Italy; terriacasonia093@gmail.com; 2 Anatomic Pathology, Department of Biomedicine and Prevention, Tor Vergata University, 00133 Rome, Italy; ferlosio@med.uniroma2.it (A.F.); scioli@med.uniroma2.it (M.G.S.); coppafrancesca@gmail.com (F.C.); 3 Cardiac Surgery Unit, Department of Surgery, Tor Vergata University, 00133 Rome, Italy; fabio.bertoldo@ptvonline.it (F.B.); lindapisano82@gmail.com (C.P.); 4 Tumor Immunology Unit, Department of Health Sciences, University of Palermo, 90134 Palermo, Italy; beatrice.belmonte@unipa.it; 5 Azienda sanitaria Provinciale di Catania (ASP), 95124 Catania, Italy; 6 Cellular and Molecular Laboratory, Department of Biomedicine, Neuroscience and Advanced Diagnostics (Bi.N.D.), University of Palermo, 90134 Palermo, Italy; carmelarita.balistreri@unipa.it

**Keywords:** abdominal aortic aneurysm, thoracic aortic aneurysm, atherosclerosis, Marfan syndrome, bicuspid aortic valve, miRNA deregulation, endothelial dysfunction, smooth muscle cell dedifferentiation, fibrosis, calcification

## Abstract

Aortic aneurysms are a serious health concern as their rupture leads to high morbidity and mortality. Abdominal aortic aneurysms (AAAs) and thoracic aortic aneurysms (TAAs) exhibit differences and similarities in their pathophysiological and pathogenetic features. AAA is a multifactorial disease, mainly associated with atherosclerosis, characterized by a relevant inflammatory response and calcification. TAA is rarely associated with atherosclerosis and in some cases is associated with genetic mutations such as Marfan syndrome (MFS) and bicuspid aortic valve (BAV). MFS-related and non-genetic or sporadic TAA share aortic degeneration with endothelial-to-mesenchymal transition (End-Mt) and fibrosis, whereas in BAV TAA, aortic degeneration with calcification prevails. microRNA (miRNAs) contribute to the regulation of aneurysmatic aortic remodeling. miRNAs are a class of non-coding RNAs, which post-transcriptionally regulate gene expression. In this review, we report the involvement of deregulated miRNAs in the different aortic remodeling characterizing AAAs and TAAs. In AAA, miRNA deregulation appears to be involved in parietal inflammatory response, smooth muscle cell (SMC) apoptosis and aortic wall calcification. In sporadic and MFS-related TAA, miRNA deregulation promotes End-Mt, SMC myofibroblastic phenotypic switching and fibrosis with glycosaminoglycan accumulation. In BAV TAA, miRNA deregulation sustains aortic calcification. Those differences may support the development of more personalized therapeutic approaches.

## 1. Introduction

The aorta is the largest arterial blood vessel in the body. It is subjected to high pressure when a large volume of blood is pumped out of the heart with each contraction, exposing it to the risk of wall degeneration and aneurysms [1]. The latter are categorized in two main types: thoracic aortic aneurysms (TAAs) and abdominal aortic aneurysms (AAAs) [2]. Aortic aneurysms are often asymptomatic until the aortic media undergoes dissection or rupture, with a high degree of morbidity and mortality [3]. Currently, the available pharmacological treatments for aortic aneurysms are not specific and aim to delay disease progression and surgical intervention [3]. TAAs and AAAs have different developmental origins as well as pathogenetic factors that induce different structural degeneration of the aortic wall [3]. Atherosclerosis and chronic inflammation are associated with AAAs [3], whereas TAAs are characterized by an increased accumulation of proteoglycans [4]. The histopathological abnormality of TAAs is named cystic medial degeneration, characterized by loss of smooth muscle cells (SMCs) and elastic fiber degeneration that leads to a weakened wall and the consequent high risk of dilatation and aneurysm formation [4,5]. Cystic medial degeneration occurs normally with aging, in particular in the presence of hypertension [3]. As mentioned above, AAAs are often associated with atherosclerosis, but as for TAAs, AAAs show SMC loss and fragmentation of elastic fibers [4]. Smoking, male sex, advanced age and atherosclerosis are mostly associated with AAAs and some sporadic (non-genetic) TAAs [1]. In fact, the majority of sporadic TAAs are mainly associated with hypertension and aging. However, there are some TAAs associated with genetic conditions, such as Marfan syndrome (MFS) and bicuspid aortic valve (BAV) [6]. All genetic TAAs occur at an early age but their underlying pathogenetic mechanisms can be very different. In fact, MFS TAAs display fibrosis [7], whereas BAV TAAs show mainly calcification [8].

## 2. Vascular Remodeling in Abdominal Aortic Aneurysms

As mentioned above, AAAs are often associated with atherosclerosis, and some evidence suggests that their formation starts from occlusive disease [9]. In particular, the formation of atheroma in the abdominal aorta starts in the subendothelial intimal space of medium- and large-sized arteries, through a multistep process. First, the inflammatory stimulus induces endothelial dysfunction and permeabilization with the consequent formation of a fatty streak. The latter evolves into an atheromatous or fibroatheromatous plaque that can rupture and provoke thrombosis [10]. During this process, endothelial cells increase the expression of vascular cell adhesion molecule-1 and intercellular adhesion molecule-1 and reduce nitric oxide synthesis [10]. The increased expression of those molecules leads to a recruitment of monocytes to the site of injury. Macrophages and T lymphocytes, in turn, express growth factors, cytokines and matrix metalloproteinases (MMPs) that stimulate the migration and proliferation of aortic SMCs into the tunica intima and the formation of atherosclerotic plaque with the accumulation of foam cells. [10]. With time, SMCs and inflammatory cells undergo apoptosis, contributing to the development of atherosclerotic lesions [11,12,13]. Crystallized calcium concentrates around the apoptotic bodies derived from dead SMCs, leading to aortic micro-calcification [3]. Moreover, AAA is characterized by a marked wall remodeling, with extracellular matrix (ECM) degeneration and destruction of elastic laminae, partially due to the increased activity of MMPs [14,15]. Currently, no association between genetic mutations and AAAs has been proven. However, genetic studies on AAA patients identified some polymorphisms in 87 genes/loci, but only 10 of them have been associated with cholesterol metabolism, atherosclerosis, inflammation and hypertension [16]. As reported above, an important risk factor for AAA is aging [17]. Among the age-related alterations of aortic SMCs that favor the progression of atherosclerosis, we found the acquisition of a stem cell phenotype [18,19]. Altogether, endothelial injury, SMC apoptosis, age-related aortic remodeling and inflammation lead to a weakened vascular wall favoring AAA development.

## 3. Phenotypic Alterations and Vascular Remodeling in Thoracic Aortic Aneurysms

In contrast to AAAs, TAAs are rarely associated with atherosclerosis and are divided into two main categories: sporadic (non-genetic) and genetic TAAs [20]. Sporadic TAAs may occur with aging, and their progression is accelerated by hypertension [20]. In young patients, TAA formation is associated with genetic conditions such as MFS and BAV.

Similarly to AAAs, TAAs are characterized by an extensive remodeling of the ECM [21]. Endothelial dysfunction is also present in TAAs, but the formation of fibro-atherosclerotic plaque is rarely evidenced [22]. In addition, endothelial cells can undergo endothelial-to-mesenchymal transition (End-MT), a process in which cells switch from an endothelial to a mesenchymal phenotype, losing cell-to-cell contact and cell polarity [23]. The main pathway responsible for the End-MT is the canonical Transforming Growth Factor β (TGF-β) signaling in association with NOTCH and Wnt/β catenin signaling [8,23,24]. End-Mt has been observed in sporadic TAAs but appears to be more accentuated in MFS TAAs [7]. At the same time, SMCs in the tunica media can dedifferentiate in myofibroblasts, switching from a contractile phenotype to a proliferative/synthetic one. Myofibroblasts deposit alcianophilic material like Glycosaminoglycans (GAGs) and collagen, determining fibrosis [25]. This process is well evidenced in sporadic TAAs and strongly exacerbated in MFS TAAs [7]. SMCs can also acquire an osteoblast-like phenotype, promoting calcification, especially in BAV TAAs [8,26]. Those alterations are associated with the deregulation of NOTCH and BMP signaling [27,28]. Moreover, ECM degeneration of the tunica media, characterized by an increased MMP activity and fragmentation and loss of elastic fibers, weakens the aortic wall such that, under high blood pressure, an aneurysm can evolve [19,29]. In the outer tunica media of TAAs, an increased number of CD4+ T lymphocytes and CD68+ macrophages as well as a large number of CD34+ and CD133+ neovessels were observed, especially in MFS TAA [21]. The increased number of structurally fragile neovessels in the outer aortic media could likely contribute to its reduced mechanical resistance with a higher risk of rupture [21]. Despite these observations, at the present time, there is no evidence of a direct association supporting a role of inflammatory cells in TAA pathogenesis [30].

## 4. miRNAs’ Biogenesis and Their Role in Aortic Remodeling

In the last few decades, many studies have demonstrated that about 97% of the human genome consists of non-coding sequences that are not transcribed in mRNAs but in non-coding RNAs [31]. Non-coding RNAs regulate gene expression at the post-transcriptional level [32]. In this manuscript, we focused our attention on the role of miRNAs in the regulation of vascular cell phenotype and aortic remodeling in TAA and AAA, as, among the non-coding RNAs, they have been the most studied in those pathologies. miRNAs are a class of non-coding RNAs, which act as epigenetic regulators without affecting the chromatin architecture [33]. In the human genome, miRNAs are encoded by introns of "host genes" and also enriched within unique miRNA clusters [33]. miRNA biogenesis starts with RNA polymerases II/III, which transcribe a primary miRNA (pri-miRNA) molecule from the intergenic or intragenic region [32]. Successively, pri-miRNAs are processed by a multi-component microprocessor complex (Drosha/DGCR8) to precursor miRNAs (pre-miRNAs) with specific hairpin structures [32]. Pre-miRNAs are exported from the nucleus to the cytoplasm via the transport factor exportin-5 using GTP as a cofactor [32]. Within the cytoplasm, pre-miRNAs are released from exportin-5 following GTP hydrolysis and are further processed by an RNase III, called “Dicer”, generating double-stranded miRNAs of approximately 22 nucleotides [32]. This product is incorporated into the RNA-induced silencing complex multiprotein complex (RISC) in which a helicase ensures that only one of the miRNA duplex strands remains in the complex to control the post-transcriptional expression of target genes [34]. miRNAs are known as negative regulators of gene expression [34]. Precisely, miRNAs induce the cleavage and degradation of target mRNAs or the inhibition of the translation process [34]. miRNAs have been demonstrated to play a critical role in several physiological processes, such as cell proliferation, apoptosis, fat metabolism, neuronal development, cell differentiation, hormone secretion and the development of multiple diseases [35]. In the last few years, many studies have reported an aberrant miRNA expression in different cardiovascular diseases such as atherosclerosis, cardiac remodeling, myocardial infarction and aneurysms [36]. In particular, it has emerged that miRNAs play a crucial role in vascular remodeling by regulating the proliferation or differentiation of SMCs and endothelial cells as well as the inflammatory or anti-inflammatory response of macrophages [31]. In this regard, emerging studies have shown that miRNAs are important regulators of the End-MT process via targeting of key components associated with End-MT signaling pathways [37,38]. Moreover, miRNAs appear to regulate the phenotypic transformation of SMCs by targeting specific genes that either participate in the maintenance of the contractile phenotype or contribute to the switch into a synthetic phenotype, thereby affecting SMC proliferation, migration, hypertrophy and differentiation [35]. Below, we describe the role of different miRNAs in the regulation of the main pathogenetic mechanisms underlying AAAs and TAAs.

## 5. The Regulatory Role of miRNAs in AAAs

Different studies have reported that inflammation, endothelial dysfunction, ECM remodeling and SMC proliferation/apoptosis, which characterize the AAA aortic wall, are associated with specific miRNA deregulations [39]. ECM proteins such as collagen and elastin are mainly produced by SMCs, and the apoptosis of those cells has been demonstrated to be implicated in the development of AAA [40]. It has been reported that SMAD3, a key intracellular mediator of the fibrotic process [41], is down-regulated in AAA [42,43]. It was also found that miR-195, an important regulator of ECM proteins, was up-regulated in AAA patients [44]. Bioinformatics analysis revealed that miR-195 has a binding site in the 3′-UTR for SMAD3, and their interaction was confirmed by in vitro studies [45]. miR-195 overexpression in vitro inhibited SMC proliferation, inducing apoptosis, whereas SMAD3 overexpression blocked those effects [45]. Therefore, miR-195 displays a crucial role in AAA pathogenesis and represents a potential therapeutic target [45].

As mentioned above, AAA is characterized by an increased degree of calcification, which can lead to rupture [46]. During AAA progression, SMCs switch from the contractile to the synthetic phenotype and synthetize osteogenic factors, such as the osteogenic transcription factor Runt-related gene (RUNX). The latter is functionally associated with SMAD2/3, which, in turn, are activated by angiotensin II (AngII) signaling [47]. The activation of the SMAD-RUNX2 signaling pathway induces osteogenic differentiation of SMCs [47]. It has been demonstrated that miR-424 has a key role in cardiovascular pathologies. miR-424 is also called “osteomir” as it regulates vascular calcification [48]. Bioinformatics analysis revealed that AAA patients express high levels of *RUNX2* and exhibit down-regulation of miR-424 compared with control subjects [47]. Other studies reported that the continuous administration of AngII to hyperlipidemic patients could induce AAA formation [49,50]. The treatment of human SMCs with AngII in vitro induced MMP overexpression through the SMAD2/3-mediated transactivation of RUNX2 [47]. In vivo treatment of ApoE KO mice with AngII showed vascular calcification, neovascularization and inflammation, whereas the treatment of ApoE KO mice with siRUNX2 mitigated AngII effects [47]. Since miR-424 is reported as an osteogenic regulator, the authors analyzed the association between that miRNA and the axis SMAD-RUNX2, proving that RUNX2 is the direct target of miR-424. In vitro treatments with siRUNX2 and miR-424 mimics reduced the activation of the SMAD-RUNX2 axis as well as the expression of factors related to AAA progression in human SMCs [47]. Those data were also confirmed in vivo by using miR-322 KO mice (miR-322 is the murine analog of miR-424). Those studies demonstrate the crucial role of miR-424 in the inhibition of aortic calcification, suggesting it as a potential therapeutic target for AAA progression [47]. miRNAs can also regulate the inflammatory response [51]. In particular, miR-33 was reported to be a key regulator of anti-inflammatory response in AAA [52]. In this light, two specific anti-microRNA oligonucleotides (AMOs) for miR-33a and miR-33b inhibition were proven to be efficient in counteracting AAA progression in vitro and in vivo [53]. First, the authors tested the efficacy of AMO on human cell lines such as macrophagic THP-1 and human SMCs. Then, in a mouse model of AAA, they inoculated AMOs for miR-33a and miR-33b inhibition. Microscopic analysis, performed on mouse AAA tissues after seven days from AMO administration, revealed a reduced number of MMP-9-positive macrophages as well as a reduced expression of monocyte chemoattractant protein-1, especially in mice treated with AMO for miR-33b [53]. Therefore, those results suggest miR-33b as a new therapeutic target to prevent AAA progression [53].

Another miRNA, reported to be involved in the chronic inflammation that characterizes the AAA aortic wall, is miR-33-5p [54]. Some studies reported that this miRNA regulates the innate immune response by the adenosine triphosphate-binding cassette transporter A1 (ABCA1) [55,56]. ABCA1 is a protein that transports free cholesterol and phospholipids from intracellular compartments to the cell membrane by using ATP as a source of energy, so this protein is fundamental in macrophage cholesterol efflux and reverse cholesterol transport [57,58]. It has been reported that ABCA1 acts as an anti-inflammatory receptor, and the enhancement of its function could be a beneficial therapeutic approach [59]. Based on those findings, Zhao et al. investigated the role of miR-33-5p in AAA progression by regulating ABCA1 [54]. Firstly, they demonstrated that aortic tissues, derived from AAA patients, showed miR-33-5p up-regulation as well as *ABCA1* down-regulation. Subsequently, using THP-1 human monocyte-derived macrophages, they confirmed *ABCA1* as a gene target of miR-33-5p [54]. Moreover, the transfection of THP-1 cells with *ABCA1* siRNA decreased the expression of p-PI3K, p-Akt. On the other hand, the transfection of THP-1 cells with miR-33-5p inhibitor restored the expression of p-PI3K, p-Akt, decreased the amount of total cellular cholesterol, promoting cholesterol efflux, and increased MMP-2, MMP-9 and TNF-α levels [54]. Therefore, the authors proved that miR-33-5p plays an important regulatory role in AAA progression and suggest its inhibition as a potential therapeutic treatment for AAA patients [54].

Another study correlated miR-21 expression to inflammatory response and aortic remodeling in AAA. Precisely, Yu et al. investigated the effects of dexmedetomidine (Dex) on miR-21 expression [60]. It has been reported that Dex suppresses the activities of inflammatory mediators and maintains a balanced myocardial function and coronary blood flow [61]. miR-21 is reported to control the inflammatory responses, ECM remodeling and lipid accumulation in cerebral aneurysms [62,63]. Moreover, it has been demonstrated that miR-21 inhibition blocks AAA development. Programmed cell death 4 (*PDCD4*), an inflammation- and apoptosis-related gene, has been proven to be a gene target of miR-21 [64,65]. In order to evaluate the effects of Dex on miR-21 expression and consequently on AAA progression, rat models of AAA were injected with Dex. The authors discovered that Dex administration in AAA rat models down-regulated the expression of inflammatory factors and MMPs as well as up-regulating miR-21 expression [60]. Moreover, *PDCD4* was confirmed to be a gene target of miR-21 also in AAA rat models. On the other hand, the combined treatment of AAA rat models with Dex and ant-miR-21 inhibited miR-21 expression and promoted AAA development [60]. Additionally, *PDCD4* inhibition reduced AAA progression and inflammatory responses [60]. In conclusion, this study proved Dex is an efficient treatment for TAA progression by miR-21 up-regulation.

## 6. The Regulatory Role of miRNAs in Sporadic TAAs

As previously reported, TAA formation is associated with progressive pathological remodeling of the aortic wall that leads to structural parietal degeneration, with rearrangement of hemodynamic loads, and finally rupture [66,67]. During this remodeling, both endothelial cells and SMCs undergo phenotypic changes in response to pathogenetic stimuli [22,68,69]. Recently, miRNA deregulation has been reported to be associated with vascular cell phenotypical changes. The phenotypic switching of SMCs from a contractile to a synthetic phenotype has been suggested to be involved in the development of aortic aneurysm and its dissection [68,69]. As already mentioned, aortic SMCs of sporadic TAA generally switch into a myofibroblast phenotype with the increased collagen synthesis and consequent aortic fibrosis [70,71]. During this process, SMCs reduced the expression of functional markers such as smooth muscle 22 α (SM22α), smooth muscle cell-specific myosin heavy chain (MYH11) and α-smooth muscle actin (α-SMA) [68]. However, the molecular mechanisms underlying the SMC phenotypic switch is not completely understood. The analysis of aortic tissues derived from patients with severe TAA evidenced a strong miR-335-5p down-regulation and Specificity Protein 1 (*SP1*) up-regulation; the latter is involved in SMC proliferation and phenotype switching [72]. However, cultured human SMCs overexpressing miR-335-5p showed *SP1* down-regulation with reduced proliferation and migration as well as increased expression of contractile markers, such as SM22α, α-SMA and CNN1 [72]. In addition, the authors documented *SP1* as a gene target of miR-335-5p. In a mouse model of aortic dissection, the administration of miR-335-5p clearly suppressed aorta dilatation and vascular media degeneration [72]. Aberrant down-regulation of miR-134-5p has been evidenced in aortic tissues derived from sporadic TAA patients [73]. In vitro studies, on aortic SMCs, revealed that miR-134-5p overexpression promoted differentiation and expression of contractile markers, suggesting a crucial role of miRNA in aortic SMC homeostasis [73]. PDGF and TGFβ are involved in SMC differentiation, vascular remodeling and aortic aneurysms [74,75]. In vitro studies demonstrated that miR-134-5p inhibits the pro-aneurysmal effects of PDGF [73]. The authors identified Signal Transducer and Activator of Transcription 5B *(STAT5B*) and integrin beta-1 (*ITGB1*), both mediators of SMC phenotypic switching, as gene targets of miR-134-5p in aortic SMCs [73]. Moreover, in a mouse model of severe AngII-induced TAA, miR-134-5p administration prevented aortic dilation and tunica media degeneration [73]. There are other miRNAs, such as the miR-29 family, that were reported to be involved in aortic SMC phenotypic switching and aneurysm formation [76]. In 18-month-old mice, the infusion with Ang-II for 1 week induced aorta dilation, increased expression of miR-29 and caused a decrease in ECM proteins [76]. The silencing of miR-29 inhibited Ang-II-induced aortic dilation and restored ECM protein expression [76]. Altogether, those studies suggest the critical role of those miRNAs in the maintenance of aortic SMC homeostasis and their representation as potential therapeutic targets to counteract aortic aneurysm progression.

Endothelial cells play a fundamental role during pathological aortic aneurysmatic remodeling [22]. Several studies identified the aberrant expression of different miRNAs in TAA and their role in the regulation of endothelial cell phenotyping and functions [77]. The activation of endoplasmic reticulum stress (ERS) contributes to the pathogenesis of cardiovascular diseases, in particular endothelial dysfunction [78,79,80]. The mechanism through which ERS mediates vascular cell dysfunction is not completely understood. miRNAs regulate the ERS response by targeting specific genes [81]. In particular, miR-204 seems to be associated with ERS by targeting sirtuin1 lysine deacetylase (*SIRT1*) [82,83]. Kassan et al. investigated the role of miR-204 in ERS and endothelial dysfunction by targeting *SIRT1* [84]. Overexpression of miR-204 in HUVECs induced ERS by up-regulation of specific stress markers, such as glucose-regulated protein, C/-EBP homologous protein and Activating Transcription Factor 6, as well as phosphorylation of PKR-like ER kinase and eukaryotic initiation factor 2. Moreover, the pharmacological treatment with external triggers of ERS up-regulated miR-204 and down-regulated *SIRT1* both in HUVECs and in mouse thoracic aorta and mesenteric resistance arteries [84]. On the other hand, miR-204 inhibition protected against the effect of ERS inductors and preserved endothelial Sirt1 levels [84]. The link between miRNA deregulation and endothelial dysfunction in sporadic TAA was also investigated. Unbiased molecular screening of miRNAs in TAA and adjacent non-aneurysmal aortas revealed ten miRNAs overexpressed in TAA tissues: miR-191-5p, miR-126-3p, miR-374-5p, miR-21-5p, miR-145-3p, miR-29c-3p, miR-133a-3p, miR-186-5p, miR-143-3p and miR-24-3p [85]. Bioinformatics analyses of miRNA gene targets displayed that some of those miRNAs are involved in different gene pathways including vascular endothelial growth factor (*VEGF*), *TGFβ* and *AKT-PI3K*. Other studies demonstrated the up-regulation and the anti-proliferative effects of miR-191 in TAA tissues, suggesting its involvement in endothelial cell senescence [85]. Although these data represent an important contribution to vascular knowledge, further functional studies are needed to describe the putative role of those deregulated miRNAs in pathogenetic mechanisms of endothelial dysfunction in sporadic TAA.

Gasiule et al. investigated the role of other miRNAs in the regulation of vascular cell phenotyping in sporadic TAA [86]. Precisely, they analyzed a panel of different miRNAs, comparing tissue and plasma samples derived from sporadic TAA patients (before and after surgery) and controls. The authors revealed different TAA-specific miRNAs in tissue and plasma samples. Among those differentially expressed miRNAs, miR-155b-5p, miR-122-3p and miR-23b-5p were able to restore their expression to normal levels after surgery, indicating their specific association with the pathology [86]. Moreover, some of those miRNAs were shown to be involved in TGF-β pathways; in particular, they are associated with SMADs and Krueppel-like factor 4 (KLF4) [86]. In fact, TAA tissues showed a marked up-regulation of KFL4, MyoCD and osteopontin; the latter is reported to be associated with VSMCs switching from the contraction to the synthetic phenotype [86]. Overall, those deregulated miRNAs are key components in TGF-β signaling and are involved in VSMC phenotypic changes, so deepening their role could be useful in identifying them as potential prognostic and therapeutic biomarkers for sporadic TAA [86].

## 7. miRNA Regulation of Vascular Cell Phenotype in Genetic TAAs

As reported above, TAAs are, in some cases, associated with genetic mutations [87]. BAV (bicuspid aortic valve) is a congenital cardiovascular malformation leading to an increased risk for severe cardiovascular events, such as TAA [88,89,90]. The BAV condition is associated with different genetic mutations including the *NOTCH1*, *TGFBR2, FBN1*, *SMAD6, GATA5* and *GATA6* genes [88,89,90]. Several studies described the differential expression of miRNAs between BAV and TAV (tricuspid aortic valve) patients [91]. Some studies reported that BAV aortopathy is also characterized by a lower expression of End-Mt markers compared with sporadic TAAs [8,92]. However, some studies demonstrated that, before dilation, BAV aortas showed an activation of the End-Mt process [93]. Using a systemic biological approach, a strong association between the miR-200 family, which targets the End-Mt transcription factors zinc-finger E homeobox-binding transcription factors 1 and 2 (*ZEB1* and *ZEB2*) [94,95], with the BAV signaling network was highlighted [93]. In particular, Maleki et al. demonstrated the involvement of miR-200c (its down-regulation) in enhancing End-Mt in non-dilated aortas of BAV patients. The authors explanted endothelial cells, the main source of miR-200c, from dilated and non-dilated aortic tissues of BAV and TAV patients. They observed, in those cells, a lower expression of miR-200c as well as the up-regulation of *ZEB1* and *ZEB2*, only in endothelial cells from non-dilated aortas of BAV patients compared with those from non-dilated TAV aortas [93]. Moreover, the authors demonstrated a negative feedback loop between miR-200c and *ZEB1* and *ZEB2*. In particular, they found a higher chromatin occupancy by *ZEB1/ZEB2* of the miR-200c promoter in BAV patients, determining miRNA down-regulation and favoring the transcription of End-Mt markers (by ZEB1/ZEB2) in non-dilated BAV aortas [93].

The differential expression of ERG and its transcription factor miR-126-5p has also been demonstrated, in the vascular phenotypic changes occurring in BAV and TAV aortas [8]. The expression levels of the protein ERG and miR-126-5p were up-regulated in TAA samples derived from BAV compared to TAV patients. Moreover, this up-regulation in BAV TAA was shown to be associated with a down-regulation of SMAD2/3 proteins [8], whose activation induces End-MT [96]. Therefore, the down-regulation of SMAD2/3 could explain the different phenotypic changes observed in the endothelium of BAV TAA. Similarly, the tunica media of BAV TAA showed an up-regulation of ERG and miR-126-5p in association with an evident aortic calcification compared with TAV tunica media; the latter were mainly characterized by a marked fibrosis [8]. Zhang et al. identified another specific miRNA that was proven to target the *SMAD2* gene [92]. The authors showed a differential expression of miR-423-5p in exosomes from BAV and TAV patients, with a significant up-regulation in BAV exosomes [92]. Prediction studies showed that miR-423-5p associates with TGF-β signaling. The latter, when activated, induces the phosphorylation of SMAD2 and SMAD3 [97]. It has been hypothesized that *SMAD2/3* could be potential targets of miR-423-5p. Transfection of human SMCs with miR-423-5p mimic induced a decreased expression of SMAD2 and p-SMAD2 [92]. Moreover, through luciferase reporter assay, the specific interaction between miR-423-5p and *SMAD2* was shown [92]. Therefore, the differential expression of miR-200, miR-126-5P and miR-423-5p in BAV TAA patients seems to be associated with the specific phenotypic changes that set it apart from sporadic TAAs.

Another familiar condition associated with a high risk of aortic complications is MFS. The latter is a rare genetic disease, characterized by mutation in the fibrillin-1 *(FBN1*) gene. FBN1 mutations lead to an impaired sequestration of latent TGF-β [98]. An important association between the hyperactivation of TGF-β signaling and the pathogenesis of MFS TAA has been reported [99]. Moreover, several studies identified TGF-β-responsive miRNAs that play a critical role in the phenotypic changes of MFS vascular cells [7,100]. Merck et al. analyzed the expression of miR-29b in the ascending aortas of MFS Fbn1^C1039G/+^ and wild-type (WT) mice [100], showing a higher expression of that miRNA in the aortas of Fbn1^C1039G/+^ mice. miR-29b is reported to regulate genes involved in apoptosis, synthesis/deposition of ECM and fibrosis [101,102,103]. In fact, the up-regulation of miR-29b was associated with increased levels of cleaved caspase-3 and caspase-9 and decreased levels of the antiapoptotic proteins Mcl-1 and Bcl-2 in the Fbn1^C1039G/+^ aortas [100]. Moreover, microscopic and biomolecular studies showed that Fbn1^C1039G/+^ aortas displayed decreased and fragmented elastin, lower expression of elastin mRNA and increased expression of MMP-2 mRNA [100]. The high expression of miR-29b was shown to be associated with a reduced activation of nuclear factor kappa-light-chain-enhancer of activated B cells (NF-KB), a repressor of miR-29b, that is suppressed by TGF-β [104]. Administration of NF-KB inhibitor to Fbn1^C1039G/+^ mice led to increased levels of miR-29b, whereas the inhibition of TGF-β or Losartan administration decreased its levels, suggesting that TGF-β1 induces miR-29b expression [100]. Finally, the administration of LNA-antimiR-29b inhibitor in Fbn1^C1039G/+^ mice has been demonstrated to prevent the effects induced by miRNA up-regulation, such as early aneurysm development, aortic wall apoptosis and ECM degeneration [100].

As mentioned above, MFS is caused by mutations in the *FBN1* gene. Some studies reported that *FBN1*–cell interaction regulates a group of miRNAs in an “outside-in” manner, influencing cell proliferation, focal adhesion and TGF-β signaling [105,106]. In this light, Zhang et al. investigated the role of fibrillin-1-controlled miRNA in the regulation of inflammatory responses and MMP12 expression in MFS pathogenesis [107]. Firstly, using fibrillin-1 hypomorphic MFS mice (Fbn1^mgR/mgR^) as severe MFS models, the authors displayed that aortic tissues derived from those mice are characterized by an increased expression of pro-inflammatory cytokines and MMPs [107]. Subsequently, they revealed that the aortic tissues of Fbn1^mgR/mgR^ mice have a strong miR-122 down-regulation as well as an increased expression of CCL2, IL-1β and MMP-12. Similar data were obtained in Fbn1^C1041G/+^ older mice [107]. In addition, aortic tissues derived from Fbn1^mgR/mgR^ mice showed a marked up-regulation of hypoxia-inducible factor 1α (HIF-1α), suggesting that MFS TAA tissues are subjected to hypoxic stress [107]. In order to find an association between a hypoxia condition and miR-122 down-regulation, human aortic smooth muscle cells (HASMCs) and ex vivo aorta cultures derived from Fbn1^mgR/mgR^ mice were subjected to hypoxic conditions. The authors demonstrated that hypoxia conditions led to an miR-122 down-regulation [107]. The treatment of HASMCs and mice aorta cultures with HIF-1α inhibitors restored miR-122 expression and reduced elastin fragmentation, inflammatory infiltration and aortic dilation. In addition, the authors revealed a molecular interaction between fibrillin-1 and miR-122 [107]. Therefore, this study proved that miR-122 down-regulation, due to fibrillin-1 deficiency and hypoxia, increases inflammatory responses and matrix remodeling in MFS TAA [107].

In other studies, miR-632 has been proven to be likely involved in MFS pathological aortic remodeling and TGF-β1 signaling [7,21]. First, a differential expression of some specific miRNAs between sporadic and MFS TAA tissues was reported, especially a very important up-regulation of miR-632 [21]. Then, by specific functional studies, it was demonstrated that miR-632 up-regulation, in MFS TAA, inhibited the DnaJ heat shock protein family (Hsp40) member B6 (*DNAJB6*) [7]. The latter is an inhibitor of the Wnt/β catenin signaling that induces the epithelial/endothelial-to-mesenchymal transition process [108]. The authors demonstrated that the down-regulation of *DNAJB6* in MFS TAA tissues led to Wnt/β catenin activation associated with End-Mt and fibrosis exacerbation [7]. TGF-β1 treatment on MFS TAA tissue fragments induced the up-regulation of miR-632 with the consequent activation of the processes mentioned above [7]. Therefore, the miR-632 seems to play a crucial role in the pathological phenotypic changes that characterize MFS TAA vascular cells [7]. Further studies are needed to assess miR-632 as a new prognostic marker and a potential therapeutic target in the progression of MFS aortopathy. A summary of all mentioned miRNAs and their roles in the regulation of vascular cell phenotype, ECM remodeling and inflammation in AAAs and TAAs is reported in Table 1.

## 8. Conclusions

**Figure 1:**
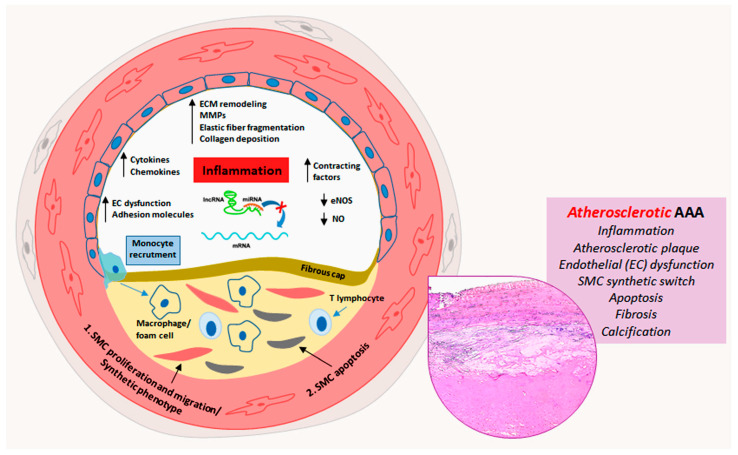
Schematic representation of miRNA deregulation-related pathogenic mechanisms in atherosclerotic abdominal aortic aneurysms. Atherosclerotic process prevails in the progression of abdominal aortic aneurysm (AAA) and is characterized at least in part by miRNA deregulation-driven endothelial dysfunction, inflammation, smooth muscle cell (SMC) proliferation, migration and apoptosis, fibroatheromatous plaque formation and thrombosis, with medial fibrosis and calcification (insert, Hematoxylin and Eosin staining).

**Figure 2:**
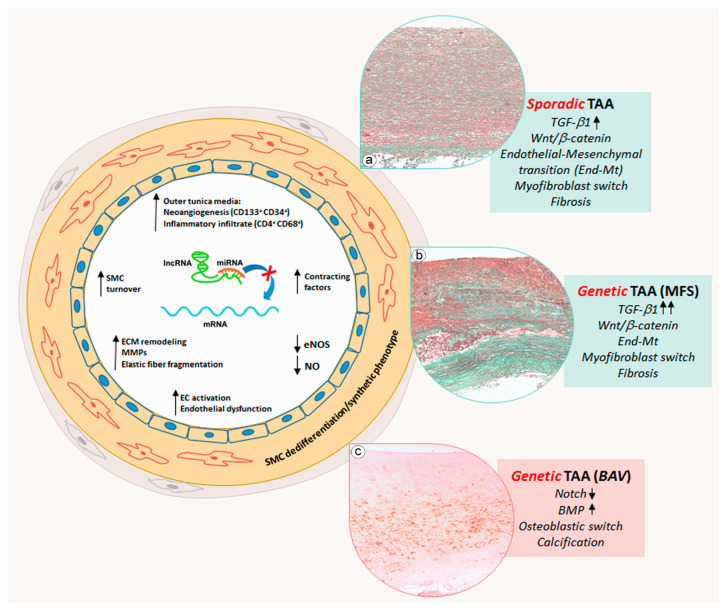
Schematic representation of miRNA deregulation-related pathogenic mechanisms in thoracic aortic aneurysms. Thoracic aortic aneurysms (TAAs) are sporadic or frequently related to genetic diseases. Sporadic and Marfan syndrome disease (MFS) TAAs show an aortic cell differentiative process, in which endothelial cells switch into a mesenchymal phenotype, whereas medial SMCs switch into a myofibroblastic phenotype and synthetize collagen and glycosaminoglycan with consequent aortic degeneration and fibrosis (inserts (**a**,**b**), Masson’s Trichrome Goldner staining). Those processes are precocious, much more marked and strongly accentuated in MFS TAAs, in which TGF-β signaling is hyperactivated. Genetic bicuspid aortic valve (BAV) TAA displays a specific degenerative process, in which SMCs dedifferentiate into osteoblast-like cells promoting medial calcification (insert (**c**), Alizarin Red staining) by miRNA deregulation-mediated activation of NOTCH and BMP signaling.

Aortic aneurysms remain a serious health concern with many clinical complications as the associated ruptures can cause significant morbidity and mortality. The onset and development of AAA and TAA are associated with different risk factors. AAA is generally associated with atherosclerosis, hypertension and aging, whereas TAA frequently occurs in patients with genetic diseases. Both types of aortic aneurysms are characterized by pathological aortic remodeling. In that light, AAA shows a more marked inflammatory parietal response compared with TAA. miRNAs have been identified to be involved in the regulation of vascular cell phenotypic transformation, inflammation and SMC apoptosis. In AAA, miR-195 and miR-21 deregulation are associated with SMC apoptosis; miR-424 down-regulation is associated with calcification of the aortic wall; miR-33b and miR-33-5p deregulation is involved in the parietal inflammatory response. In TAA, miRNA deregulation induces a vascular cell phenotype switch. In sporadic TAA, miR-335-5p, miR-134, miR-155b-5p, miR-122-3p and miR-23b-5p down-regulation lead to a reduction in SMC cytoplasmic contractile cytoskeletal filaments with a switch into a synthetic phenotype promoting medial fibrosis. In TAA, endothelial cells characteristically lose physiological endothelial markers, with an End-Mt phenotypic switch and consequent endothelial dysfunction. For genetic TAA, MFS TAA shares histopathological features with sporadic TAA such as End-Mt and fibrosis, but in a more accentuated manner. Deregulation of specific miRNAs such as miR-29, miR-122 and miR-632 plays a key role in the regulation of the phenotypical cell changes and parietal remodeling observed in MFS TAA. Aortic remodeling in BAV TAA differs from sporadic and MFS TAAs, and miRNAs appear crucial in the regulation of aortic remodeling. Deregulation of miR-126, miR-200 and miR-423-5p seems to inhibit the End-Mt process and favor the calcification of the tunica media observed in BAV TAA. Schematic representations of the phenotypic alterations and aortic remodeling of AAA and TAA regulated by miRNAs are shown in Figure 1 and Figure 2. Overall, miRNA deregulation has an important impact in inducing inflammation and apoptosis in AAAs as well as in stimulating endothelial-cell and SMC phenotypic switching in TAAs (sporadic and genetic). A better understanding of deregulated miRNAs and related gene pathways may provide precious information for the development of miRNA-based therapies aimed at preventing aortic aneurysm progression. Furthermore, the evaluation of circulating miRNAs and their correlation with the disease severity could represent a potential prognostic value.

## Figures and Tables

**Table 1:** Deregulated miRNAs involved in vascular phenotypic changes and aneurysmatic aortic remodeling.

miRNA	Cell Type	Gene Target/ Pathway Associated	Pathology	miRNA Deregulation	Aortic Effects	Reference
miR-195	SMCs	SMAD3	AAA	↑	SMC apoptosis, parietal remodeling	[45]
miR-424	SMCs	RUNX2	AAA	↓	Calcification	[47]
miR-33b	SMCs, macrophages	Inflammatory signaling	AAA	↑	Inflammation	[53]
miR-33-5p	THP-1 human monocyte-derived macrophages	ABCA1	AAA	↑	Inflammation, inhibition of cholesterol efflux	[54]
miR-21	ECs and VSMCs	PDCD4	AAA	↓	Inflammation, apoptosis	[60]
miR-335-5p	SMCs	SP1	Sporadic TAA	↓	Proliferation, migration and switch into a synthetic phenotype	[72]
miR-134-5p	SMCs	STAT5B and ITGB1	Sporadic TAA	↓	Migration and increased switch into a synthetic phenotype	[73]
miR-29 family	SMCs	Angiotensin II signaling	Sporadic TAA	↑	Parietal remodeling	[76]
miR-204	ECs	Sirt1	Sporadic TAA	↑	Increased ERS/ dysfunction	[84]
miR-191	ECs	CDK6, SATB1 (putative)	Sporadic TAA	↑	Increased cell senescence/ dysfunction	[85]
miR-155b-5p, miR-122-3p and miR-23b-5p	VSMCs	SMAD, KFL4	Sporadic TAA	↓	VSMC switch from contractile to synthetic phenotype	[86]
miR-200c	ECs	ZEB1 and ZEB2	BAV ND	↓	Increased End-Mt	[93]
miR-126-5p	ECs/SMCs	SMAD2/3 (putative)	BAV TAA	↑	Inhibition of End-Mt/ calcification	[8]
miR-423-5p	SMCs	SMAD2	BAV TAA	↑	Reduced fibrosis	[92]
miR-29b	SMCs	TGF-β/ NFκB signaling	MFS TAA	↑	Increased apoptosis, ECM deficiencies, remodeling	[100]
miR-122	VSMCs	FBN-1/ hypoxia	MFS TAA	↓	Inflammation and matrix remodeling	[107]
miR-632	ECs/ SMCs	DNAJB6	MFS TAA	↑	Increased End-Mt and fibrosis	[7]

Abbreviations: SMCs, smooth muscle cells; AAA, abdominal aortic aneurysm; ↑, up-regulaton;RUNX2, RUNX family transcription factor 2; ↓, down-regulation;ABCA1, adenosine triphosphate-binding cassette transporter A; PDCD4, programmed cell death 4; SP1, Specificity Protein 1; TAA, thoracic aortic aneurysm; STAT5B, Signal Transducer and Activator of Transcription 5B; ITGB1, integrin beta-1; ECs, endothelial cells; Sirt1, sirtuin1 lysine deacetylase; ER, endoplasmic reticulum; CDK6, cell division protein kinase 6; SATB1, Special AT-rich Sequence Binding Protein 1; KFL4, Krueppel-like factor 4; ZEB1 and ZEB 2, zinc-finger E homeobox-binding transcription factors 1 and 2; BAV, bicuspid aortic valve; ND, non-dilated; End-Mt, endothelial–mesenchymal transition; TGF-β, Transforming Growth Factor β; NFκB, nuclear factor kappa-light-chain-enhancer of activated B cells; MFS, Marfan syndrome; ECM, extracellular matrix; DNAJB6, DnaJ heat shock protein family (Hsp40) member B6.
